# 中线癌发病机制及治疗的研究进展

**DOI:** 10.3779/j.issn.1009-3419.2024.101.16

**Published:** 2024-06-20

**Authors:** Jiaxin DU, Xinwei ZHANG

**Affiliations:** 300060 天津，天津医科大学肿瘤医院生物治疗科，国家恶性肿瘤临床医学研究中心，天津市肿瘤防治重点实验室，天津市恶性肿瘤临床医学研究中心，天津市肿瘤免疫与生物治疗重点实验室; Department of Immunology, Tianjin Medical University Cancer Institute and Hospital, National Clinical Research Center for Cancer, Tianjin Key Laboratory of Cancer Prevention and Therapy, Tianjin’s Clinical Research Center for Cancer, Key Laboratory of Cancer Immunology and Biotherapy, Tianjin 300060, China

**Keywords:** 中线癌, BRD4, NUTM1, 发病机制, 治疗, NUT carcinoma, BRD4, NUTM1, Pathogenesis, Treatment

## Abstract

中线癌（nuclear protein in testis carcinoma），又称NUT癌，是一种罕见的高度侵袭性恶性肿瘤，最常见于中线器官和肺。NUT癌的特征性遗传学改变是NUT癌家族成员1（NUT midline carcinoma family member 1, NUTM1）基因重排。本文将对其最常见的融合形式溴结构域蛋白4（bromodomaincontaining protein 4, BRD4）-NUTM1融合基因的致病机制以及靶向药物研发的进展进行综述。

## Abstract (en)

NUT carcinoma (nuclear protein in testis carcinoma) is a rare and highly invasive malignant tumor, which is most common in midline organs and lungs. The characteristic genetic change of NUT carcinoma is the rearrangement of NUT middle carcinoma family member 1 (NUTM1) gene. In this article, we will review the pathogenic mechanism of its most common fusion form, bromodomaincontaining protein 4 (BRD4)-NUTM1 fusion gene, and the progress in the research and development of targeting drugs.

中线癌（nuclear protein in testis carcinoma），又名NUT癌，该病于1991年首次被报道，常发生于头颈部及纵隔等人体中线结构，因此命名为中线癌^[1]^。NUT癌具有高度侵袭性，在诊断时通常已处于晚期，化疗效果不佳，中位生存期为6.7-9.5个月^[2]^。NUT癌家族成员1（NUT midline carcinoma family member 1, NUTM1）基因重排是NUT癌特征性改变，也可见于鼻癌^[3⇓⇓-6]^、肝癌^[7]^、下颌下腺^[8]^、肉瘤样肿瘤^[9]^、汗孔瘤^[10]^和梭形细胞癌^[11]^等肿瘤，统称为NUTM1重排肿瘤。最初发现NUT癌由NUTM1与含溴结构域蛋白4（bromodomain-containing protein 4, BRD4）融合伴侣基因发生融合，产生BRD4-NUTM1嵌合基因，后发现NUTM1和其他伴侣基因（如BRD3^[12]^、NSD3^[13]^、MGA^[9,14]^等）发生融合，统称为NUT变体（NUT-variant）^[15]^。75%的NUT癌患者存在t（15; 19）（q14; p13.1）形成的BRD4-NUTM1融合基因，并发现其预后差于NUT变体^[15]^。用小干扰RNA（small interfering RNA, siRNA）敲除 NUT癌细胞系中BRD4-NUTM1基因表达后，癌细胞快速鳞状分化且生长受到抑制^[16]^，并且，仅BRD4-NUTM1融合基因即可诱导小鼠发生肿瘤^[17]^，表明BRD4-NUTM1融合蛋白是NUT癌潜在的治疗靶点。

## 1 NUT癌的关键因子

### 1.1 MUTM1基因

NUTM1位于染色体15q14，编码1132个氨基酸，含有核定位和核输出信号，使其能够在核和细胞质之间穿梭^[18]^。生理情况下，MUTM1在男性生殖细胞中表达，招募组蛋白乙酰转移酶（histone acetyl transferase, HAT）p300和/或环磷酸腺苷反应元件结合蛋白的结合蛋白（cAMP responsive element binding protein binding protein, CBP）并使组蛋白H4K5和H4K8乙酰化^[19]^，参与减数分裂后的转录抑制。NUTM1基因重排保留了NUT高度保守的酸性结构域（acidic domain 1, AD1），能够结合和激活HAT p300^[20]^。需要注意的是，NUTM1基因的融合伴侣也参与了NUTM1融合蛋白的功能。

### 1.2 BRD4

BRD4和BRD2、BRD3以及BRDT属于BET蛋白家族成员，该蛋白家族包含两个乙酰化组蛋白结合溴结构域和一个额外末端蛋白质相互作用结构域。该蛋白家族可以识别组蛋白Kac修饰的表观遗传信息，从而调控基因表达、重塑染色质结构和防止复制应激诱导的DNA损伤^[21]^。当BRD2/3/4与NUTM1发生融合时，可产生编码融合蛋白的BRDX-NUTM1嵌合基因。BRD4-NUT融合蛋白保留野生型BRD4与P-TEFb（CDK9/CyclinT1）等蛋白的相互作用^[22]^，可显著促进靶基因的转录水平，最终导致原癌基因激活，提示CDK9是潜在的治疗靶点。

## 2 NUT癌的发病机制

### 2.1 BRD4-NUT融合蛋白在发病机制中的作用

BRD4-NUT融合蛋白通过BRD4的一个疏水溴结构域与组蛋白尾部乙酰赖氨酸上的染色质结合，并凝集于染色质外^[23]^，还利用NUT蛋白F1c结构域的两个N端基序（377-418; 419-470）结合p300-TAZ2^[20]^，激活p300蛋白质并使其附近的组蛋白末端乙酰化，进而招募更多的BRD4-NUT蛋白质，最终形成染色质局部的BRD4-NUT/p300高度富集区域，即组蛋白超级乙酰化结构域^[20]^。这种富集BRD4-NUT、野生型BRD4、p300和活性组蛋白标记[包括H3K27Ac，不包括抑制性（H3K27Me3）标记]的大量基因组区域（100 kb-2 MB），被称为“megadomains”（MD）^[24]^。MD通过类似正反馈的过程从一个位点进行性扩展，逐渐扩大形成成熟的MD^[24]^。这种类MD大小可能受p300的数量^[25]^和组蛋白去乙酰化酶（histone deacetylase, HDAC）复合物的影响。而p300在核内的数量是有限的，向MD的募集可能会降低其在其他基因座的丰度，使MD在增加靶区域转录的同时抑制非靶区域的转录，双向影响NUT癌细胞的生长和分化。因此，通过拮抗p300，或采用HDAC抑制剂阻断MD形成，进而抑制BRD4-NUT的活性成为NUT 癌治疗的潜在靶点。而MD在结构和功能上与超级增强子高度相似，它们的位置在不同的细胞系和组织中不同，且都可以通过转录共激活因子将转录因子连接到启动子来激活RNA聚合酶II^[24]^。此外，MD还被发现形成一种转录独立的新的第六亚室，称为亚室M（subM）^[26]^，其内包含常见的MD相关TF、MYC、SOX2和TP63，可以在相对染色臂和不同染色体之间形成远距离的MD-MD相互作用，使其在功能和结构上相互协调。

### 2.2 BRD4-NUT通过MYC、TP63和SOX2基因驱动癌症的发生

BRD4-NUT增强MYC的表达，包括CCAT1和PVT1，上调和激活CDK4/6和细胞周期蛋白D1来促进癌症生长。实验^[24]^证明，敲低PVT1或MYC可导致NUT癌细胞的分化。大多数NUT癌中表达TP63，TP63是一种与TP53相关的鳞状细胞干细胞因子和肿瘤抑制因子，敲低TP63可以有效地诱导NUT癌细胞的死亡^[24]^。SOX2是一种参与维持细胞干细胞特性的转录因子，其过表达可以驱动鳞状细胞癌，敲低SOX2可导致NUT癌细胞的分化^[27]^。SOX2和MYC可以协作以阻断分化并维持NUT癌的干细胞样状态^[28]^。SOX2和p63可能共同驱动鳞状细胞特异性程序促进NUT癌细胞的生长^[29]^。

### 2.3 BRD4-NUT上调ALX1促进NUT癌细胞迁移

BRD4-NUT蛋白可通过NUT F1c结构域在ALX1基因的启动子区域异常激活p300的活性并参与形成超级乙酰化区域（H3K27ac），从而凝集BRD4长亚型（bromodomain-containing protein 4-long, BRD4-L）蛋白等转录机器相关蛋白复合物，上调ALX1基因表达水平，最终激活转录因子Snail蛋白介导的上皮间充质转化通路，促进NUT癌细胞迁移^[20]^。阻断BRD4-NUT蛋白与p300之间的相互作用，可以抑制上皮间充质转化通路和细胞侵袭，改善预后，成为NUT癌治疗的潜在靶点。

## 3 NUT癌的治疗

NUT癌侵袭性较高，预后较差，症状不典型，故在诊断和鉴别诊断上存在困难，诊断分期较晚，目前还没有标准治疗方案。传统治疗方法中，早期放疗和手术可以提高患者生存率^[30,31]^，化疗则无明显作用，NUT癌对化疗反应率约为40%，且无论使用哪种化疗药物类型都容易耐药^[32]^。由于NUT癌细胞具有鳞癌分化倾向，因此一线治疗多选择鳞状上皮细胞癌相关的化疗方案，并兼顾NUT癌原发部位的特点实施个体化治疗。NUT癌是一种低肿瘤突变负荷的冷肿瘤，对免疫疗法不敏感^[33]^，但部分患者接受放疗加免疫联合治疗后可获得更长的生存期^[34⇓-36]^，故应用前可行程序性细胞死亡配体1（programmed cell death ligand 1, PD-L1）表达等检测预测疗效。

随着NUT癌致病机制研究的进一步深入，更多的学者将希望给予靶向治疗方案，多种NUT癌靶向药物已进入临床试验阶段，具体包括：BET抑制剂、p300抑制剂、HDAC抑制剂、CDK9抑制剂。

### 3.1 BET抑制剂

BET抑制剂可在溴结构域‐染色质相互作用位点与组蛋白尾部的乙酰化裂解素竞争来阻断BRD4蛋白与染色质的结合，从而抑制参与M和G_1_期的基因转录，并影响有丝分裂，诱导鳞状分化及抗增殖。且对于非BRD4-NUTM1融合的NUT癌，BET抑制剂也具有令人期待的治疗效果。RO6870810（RG 6146，以前称为TEN-010）是一种新型小分子、非共价BET蛋白抑制剂，含有与BET家族BRD相互作用的相同核心噻吩并二氮杂卓支架，且具有高亲和力，目前已完成I期研究^[37]^，小分子BET抑制剂Molibresib（GSK 525762）^[38]^和BET抑制剂BMS-986158^[39]^也已完成I期研究，具体研究进展见表1。

**表1:** BET抑制剂研究进展

Project	RO6870810	Molibresib	BMS-986158
Dosage	0.65 mg/kg	80 mg	Range: 0.75-4.5 mg
Administration	Once a day by subcutaneous injection	Orally once a day	5 d on, 2 d off
Sample size	8	19	46
PR	25% (n=2)		4% (n=2)
SD	63% (n=5)	42% (n=8)	26% (n=12)
PD	13% (n=1)	21% (n=4)	52% (n=24)
Median PFS	94 d (range: 15-783 d)	75 d	58 d (range: 50-68 d)
Common treatment-related adverse events	Fatigue (42%), decreased appetite (35%) and injection-site erythema (35%)	Thrombocytopenia (51%), gastrointestinal events (22%-42%), anemia (22%) and fatigue (20%)	Diarrhea (43%) and thrombocytopenia (39%)

PR: partial response; SD: stable disease; PD: progressive disease; PFS: progression-free survival.

BET抑制剂不能完全抑制BRD4-NUT的功能，且会影响正常细胞活性，同时NUT癌细胞可以通过MYC上调细胞周期蛋白D1绕过BET抑制诱导的生长停滞^[40]^，故BET抑制剂单一疗法的作用有限且易产生耐药，联合治疗是未来研究方向，如BET抑制剂与HAT抑制剂A485联用^[41]^，以及新型BET-p300双溴域抑制剂NEO2734方案^[42]^值得期待。

### 3.2 p300抑制剂

 p300抑制剂的研发主要聚焦于其BRD结构域和HAT结构域，如BRD结构域抑制剂GNE-781^[43]^、HAT结构域高选择性抑制剂A-485^[44,45]^。TAZ2结构域在BRD4-NUT蛋白招募p300蛋白的过程中起关键作用^[20]^，或可成为NUT癌潜在的治疗靶点。

### 3.3 HDAC抑制剂

HDAC抑制剂耗尽的巨型结构域包括支持MYC和SOX2表达的靶向区域，这两者在阻断分化和维持NUT癌生长中起着关键的致癌作用。HDAC参与染色体结构修饰和基因表达调控，并发挥重要作用，HDAC抑制剂可增加染色体乙酰化的程度，阻断细胞周期调控以及细胞分裂的关键基因转录激活，从而逆转BRD4-NUT功能，恢复细胞分化，发挥抗肿瘤活性。1例11岁唾液腺NUT癌患儿在使用HDAC抑制剂Vorinostat后获得了长期的生存期^[46]^，实验证明HDAC抑制剂Panobinostat也有延长生存期的作用^[47]^，一种双PI3K/组蛋白去乙酰化酶抑制剂CUDC‐907（NCT02307240）已进入I期研究，其安全性和初步抗癌活性的结果暂未公布^[48]^。

### 3.4 CDK9抑制剂

CDK9与CyclinT1一起形成P-TEFb复合物，并通过过度磷酸化RNA聚合酶II诱导转录激活，CDK9抑制剂通过诱导NUT癌细胞的凋亡和DNA损伤反应来特异性调节转录延伸并有效损害靶基因活力^[49]^，使得CDK9成为一个敏感的靶点。CDK抑制剂Flavopiridol可以在NUT癌细胞中抑制Mcl-1、诱导DNA损伤应答和细胞凋亡，在体外实验和动物实验中均延缓了体内肿瘤生长^[50]^。

## 4 总结

NUT癌是一种极具侵袭性的恶性肿瘤，因其恶性程度高、发病率低、症状非特异性，导致诊断和鉴别诊断困难和传统治疗手段效果不佳。本文对NUT癌及其发病机制和治疗的研究进展进行了综述，对目前的治疗方案和药物研究进展进行了阐述，文中讨论了BET抑制剂、p300抑制剂等药物的治疗方案和不良反应，并探讨了NUT癌潜在的治疗靶点。相信对NUT癌发病机制研究的不断深入和新的治疗靶点及用药方案的合理化，可以为临床医生的诊疗和鉴别提供新的思路，也为患者带来希望。
